# Mutational pattern and frequency of induced nucleotide changes in mouse ENU mutagenesis

**DOI:** 10.1186/1471-2199-8-52

**Published:** 2007-06-20

**Authors:** K Ryo Takahasi, Yoshiyuki Sakuraba, Yoichi Gondo

**Affiliations:** 1 Population and Quantitative Genomics Team, RIKEN Genomic Sciences Center, Yokohama 230-0045, Japan

## Abstract

**Background:**

With the advent of sequence-based approaches in the mutagenesis studies, it is now possible to directly evaluate the genome-wide pattern of experimentally induced DNA sequence changes for a diverse array of organisms. To gain a more comprehensive understanding of the mutational bias inherent in mouse ENU mutagenesis, this study describes a detailed evaluation of the induced mutational pattern obtained from a sequence-based screen of ENU-mutagenized mice.

**Results:**

Based on a large-scale screening data, we derive the sequence-based estimates of the nucleotide-specific pattern and frequency of ENU-induced base replacement mutation in the mouse germline, which are then combined with the pattern of codon usage in the mouse coding sequences to infer the spectrum of amino acid changes obtained by ENU mutagenesis. We detect a statistically significant difference between the mutational patterns in phenotype- versus sequence-based screens, which presumably reflects differential phenotypic effects caused by different amino acid replacements. We also demonstrate that the mutations exhibit strong strand asymmetry, and that this imbalance is generated by transcription, most likely as a by-product of transcription-coupled DNA repair in the germline.

**Conclusion:**

The results clearly illustrate the biased nature of ENU-induced mutations. We expect that a precise understanding of the mutational pattern and frequency of induced nucleotide changes would be of practical importance when designing sequence-based screening strategies to generate mutant mouse strains harboring amino acid variants at specific loci. More generally, by enhancing the collection of experimentally induced mutations in unambiguously defined genomic regions, sequence-based mutagenesis studies will further illuminate the molecular basis of mutagenic and repair mechanisms that preferentially produce a certain class of mutational changes over others.

## Background

In the post-sequencing era of genome biology, the main focus of interest has shifted from questions of genome structure to problems of gene function. Many research activities are now directed towards establishing precise understanding of genetic architecture underlying phenotypic variation. Recently, chemical mutagenesis has become a major method of promoting functional analysis of the mouse genome. *N*-ethyl-*N*-nitrosourea (ENU) is known to generate a spectrum of alleles, mainly by introducing single base replacement changes (i.e. transitions and transversions), and has been the mutagen of choice for enhancing the genetic resource for biomedical applications [1,2].

A central issue of any mutagenesis screen is its efficiency in recovering mutations of interest. One criticism concerning the utility of ENU mutagenesis is that it may not be an efficient way of producing appropriate models of naturally occurring genetic variants (such as human genetic disorders) [3]. Indeed, ENU-induced nucleotide changes are known to exhibit a strong bias towards particular lesions [4,5], and possibilities remain that this property should restrict the general applicability of ENU mutagenesis.

While phenotype-based mutagenesis screens have been effective in obtaining a variety of phenotypic mutants [6], ENU mutagenesis has recently been extended to gene-driven analyses of the mouse genome aimed at producing allelic series of functional mutations at particular loci [7-14]. The sequence-based approach is also suited for analyzing the mechanisms of mutation, as it should identify any sequence changes induced within the targeted regions, thereby allowing a precise evaluation of the true mutational pattern. To gain a more comprehensive understanding of the mutational bias inherent in ENU mutagenesis, this article describes a detailed examination of the pattern of induced DNA sequence changes recently obtained from our sequence-based screen of ENU-mutagenized mice using a temperature gradient capillary electrophoresis (TGCE) system [13]. Our aim here is four-fold: (i) to estimate the frequency per nucleotide site of ENU-induced mutation; (ii) to see if there is any indication of strand-specific pattern of induced mutation; (iii) to contrast the mutational patterns in phenotype- versus sequence-based mutagenesis screens; and (iv) to infer the spectrum of amino acid changes obtained by ENU mutagenesis screens. The new data provided by the present analysis will be of great use in promoting the efficient production of murine models for human genetic disorders. Moreover, it will also expand our understanding of molecular mechanisms of mutagenic processes, which are the underpinnings of any comparative and evolutionary genomic data.

## Results and discussion

### Site-specific pattern and frequency of induced mutations

Whereas our mutagenesis screen includes nongenic portion of the mouse genome [13], the present analysis focuses on the sequence changes identified within the protein-coding genes (or more specifically, mutations detected in exonic as well as flanking intronic or promoter regions) so that the detected mutations are classified as base changes on the nontranscribed (i.e. sense) strand. Excluding those mutations detected in the intergenic sequences, we have identified, by scanning a total of 181,031,647 nucleotide sites, 131 unique germline mutations, which include 130 base replacement changes as summarized in Table 1; the remainder is a length mutation that deletes a cytosine site on the nontranscribed strand. The overall frequency of base replacement mutation is therefore obtained as 7.18 × 10^-7 ^per nucleotide site per generation. (Inclusion of mutations detected in intergenic sequences yields a slightly higher rate of 7.49 × 10^-7 ^[13].) Based on a preliminary experiment utilizing Taq polymerase-induced errors as positive controls, we determined that under the experimental conditions used in our mutation screening [13], the rate of mutation discovery by our TGCE system was ~50%; the absolute rate of ENU-induced heritable mutation is therefore estimated roughly as 1.4 × 10^-6 ^per nucleotide site, which is approximately two orders of magnitude higher than the spontaneous mutation rate in humans (~2 × 10^-8 ^per site per generation [15-17]). The detail of the control experiment will be published elsewhere.

**Table 1:** Frequency of base replacement changes in the sequence-based screen

Base change				
	
From	To	*k*	*L*	μ × 10^7 ^(95%CI)
T	C	31		6.49 (4.58 – 9.21)
	A	21		4.39 (2.88 – 6.72)
	G	5		1.05 (0.46 – 2.44)
Subtotal		57	47,799,305	11.92 (9.21 – 15.45)
C	T	18		4.24 (2.69 – 6.70)
	A	6		1.41 (0.66 – 3.07)
	G	0		0.00 (0.00 – 0.71)
Subtotal		24	42,475,136	5.65 (3.81 – 8.41)
A	T	8		1.73 (0.89 – 3.40)
	C	2		0.43 (0.13 – 1.56)
	G	18		3.89 (2.47 – 6.14)
Subtotal		28	46,311,939	6.05 (4.20 – 8.74)
G	T	6		1.35 (0.63 – 2.94)
	C	1		0.22 (0.05 – 1.25)
	A	14		3.15 (1.89 – 5.29)
Subtotal		21	44,445,267	4.72 (3.10 – 7.22)

Total		130	181,031,647	7.18 (6.05 – 8.53)

Mutations are viewed as base changes on the nontranscribed strand.

Table 1 also shows that in accord with the previous observations [1,18], ENU preferentially introduced heritable changes at T/A sites. In contrast, only a single instance of G-to-C transversion was recorded, while C-to-G transversion was not detected at all. Although there are several reported instances of putatively ENU-induced G/C-to-C/G changes (e.g. [7,11,19,20]), we suspect that those mutations are in fact spontaneous.

Our collection of ENU-induced mutations further suggests that the mutation frequency at thymines on the nontranscribed strand is substantially higher than the frequency at adenines (11.92 versus 6.05 mutations among 10^7 ^sites; Table 1). The 95% confidence intervals (CIs) do not overlap with each other, suggesting that the observed strand asymmetry is not due merely to chance events but instead reflects the true nature of strand-specific mutagenic mechanisms; heritable changes are predominantly introduced at T/A base pairs when thymines are located on the nontranscribed strand (χ^2^_df = 1 _= 9.001, *P *= 0.003). Strand-specificity was not detected among changes at G/C pairs (5.65 versus 4.72 mutations among 10^7 ^sites; χ^2^_df = 1 _= 0.359, *P *= 0.549). The possible cause of the mutational asymmetry will be discussed later in this section.

### Consequences of ENU mutagenesis on amino acid sequences

Since the distribution of ENU-induced base replacement changes (as described in Table 1) is markedly different from the pattern of spontaneous mutation [15-17], the utility of mouse ENU mutagenesis in recovering disease-causing variants may somehow be limited, solely because it produces a particular class of amino acid changes more effectively than others. To see if the skewed distribution of ENU-induced mutation should lead to biased generation of amino acid variants, we here compute the expected distribution of amino acid replacement changes in the ENU-mutagenized mouse proteome, as detailed in the Methods section below.

Among 420 (= 21 × 20) types of amino acid replacement changes (including those involving termination codons), 170 are possible through single base replacement, whereas other changes necessarily require multiple mutational steps. By combining the per-site frequency of ENU-induced base replacement mutation (Table 1) with the pattern of codon usage in the mouse coding sequences [21], the relative frequencies of the 170 possible amino acid changes were obtained as shown in Table 2, where the expected count of each change among 1,000 nonsynonymous mutations is listed. Expected proportion of synonymous versus nonsynonymous changes for each amino acid was also obtained as summarized in Table 3, which shows that for certain amino acids (e.g. alanine, glycine, and proline), more than 40% of induced base replacement mutation result in synonymous changes. Overall, it is found that 71.5% of base replacement mutations would result in nonsynonymous changes (67.4% missense, 3.9% nonsense, and 0.2% make-sense), while the remaining 28.5% would lead to synonymous changes that do not alter amino acid sequences. As we shall see below, this theoretical prediction is highly concordant with the observed pattern of synonymous and nonsynonymous mutations in the sequence-based screens.

**Table 2:** Expected frequency of amino acid replacement changes

	To																					
		
From	Ala	Arg	Asn	Asp	Cys	Gln	Glu	Gly	His	Ile	Leu	Lys	Met	Phe	Pro	Ser	Thr	Trp	Tyr	Val	X	Total
Ala	*			4.4			2.1	0.0							1.0	6.3	14.6			19.7		48.3
Arg		*			4.1	3.6		6.2	3.0	1.1	2.8	5.0	1.1		0.5	4.3	0.4	4.4			3.3	39.7
Asn			*	9.5					1.1	4.2		7.6				9.5	1.1		4.2			37.0
Asp	1.4		10.1	*			10.3	12.5	0.7										4.3	5.5		44.9
Cys		10.1			*			1.6						2.1		7.2		0.8	4.9		4.4	31.2
Gln		12.0				*	0.0		5.3		5.3	4.4			1.3						13.1	41.5
Glu	1.9			8.2		1.0	*	17.5				14.2								7.8	6.1	56.7
Gly	1.0	7.8		7.0	3.0		6.8	*								7.0		1.4		5.9	1.5	41.6
His		6.7	2.4	0.0		5.2			*		3.0				0.7				7.3			25.4
Ile		0.5	11.4							*	2.2	2.1	3.0	4.5		2.7	19.9			11.9		58.0
Leu		5.7				14.2			9.9	5.9	*		7.7	11.9	35.6	8.7		0.9		1.4	6.3	108.3
Lys		14.6	6.8			1.6	14.6			2.5		*	4.0				1.6				6.5	52.2
Met		1.6								7.3	3.3	6.8	*				10.0			6.0		35.1
Phe					2.8					11.6	25.4			*		17.1			11.6	2.8		71.1
Pro	0.0	0.0				2.2			3.5		17.2				*	17.2	5.7					45.8
Ser	3.5	7.4	6.8		3.7			8.4		2.9	4.5			9.8	21.9	*	15.3	0.0	3.3		1.5	89.1
Thr	14.2	0.0	3.1							13.9		2.1	1.6		1.6	6.3	*					42.9
Trp		9.1			1.3			0.9			1.1					0.2		*			5.2	17.8
Tyr			8.4	2.0	7.4				12.4					3.3		0.8			*		6.0	40.4
Val	27.3			7.8			10.7	4.4		7.1	4.2		6.1	2.4						*		70.0
X		0.9			0.2	0.6	0.1	0.1			0.3	0.4				0.1		0.5	0.2		*	3.2

Total	49.4	76.5	49.0	38.8	22.5	28.5	44.7	51.6	35.9	56.5	69.4	42.5	23.5	33.9	62.7	87.3	68.6	8.0	35.7	61.0	54.0	1000.0

Frequencies are adjusted so as to give the expected number of changes per 1,000 nonsynonymous mutations. Blanks correspond to amino acid replacements that require multiple mutational steps. An X designates termination codons, whereas asterisks (*) indicate synonymous changes.

**Table 3:** Amino acid-specific pattern of synonymous and nonsynonymous changes

	To																				
	
From	Ala	Arg	Asn	Asp	Cys	Gln	Glu	Gly	His	Ile	Leu	Lys	Met	Phe	Pro	Ser	Thr	Trp	Tyr	Val	X
Ala	*41.9*			5.3			2.6	0.0							1.3	7.6	17.6			23.7	
Arg		*35.0*			6.7	5.9		10.1	5.0	1.7	4.7	8.2	1.8		0.8	7.1	0.6	7.2			5.3
Asn			*25.5*	19.0					2.1	8.5		15.4				19.0	2.1		8.5		
Asp	2.2		16.4	*27.3*			16.7	20.2	1.2										7.0	9.0	
Cys		25.6			*21.0*			4.1						5.3		18.3		2.0	12.4		11.2
Gln		23.2				*19.9*	0.0		10.3		10.3	8.4			2.6						25.3
Glu	2.7			11.3		1.4	*21.5*	24.2				19.7								10.8	8.4
Gly	1.4	11.0		9.9	4.3		9.6	*41.3*								9.9		2.0		8.4	2.2
His		19.5	7.1	0.0		15.3			*25.9*		8.7				2.2				21.3		
Ile		0.6	14.4							*26.6*	2.7	2.7	3.7	5.6		3.4	25.1			15.1	
Leu		3.4				8.4			5.8	3.5	*36.1*		4.6	7.0	21.0	5.1		0.6		0.8	3.7
Lys		22.4	10.4			2.5	22.4			3.9		*19.8*	6.1				2.5				10.0
Met		4.6								20.8	9.5	19.4					28.6			17.1	
Phe					3.2					13.6	29.9			*16.2*		20.1			13.6	3.2	
Pro	0.0	0.0				2.9			4.6		22.4				*40.2*	22.4	7.5				
Ser	2.8	5.9	5.4		3.0			6.7		2.3	3.6			7.7	17.4	*29.3*	12.1	0.0	2.6		1.2
Thr	20.5	0.0	4.5							20.0		3.0	2.4		2.3	9.1	*38.2*				
Trp		50.9			7.4			4.9			6.3					1.1					29.5
Tyr			16.7	4.0	14.8				24.7					6.6		1.6			*19.8*		11.9
Val	28.2			8.0			11.1	4.6		7.4	4.4		6.3	2.5						*27.6*	
X		23.1			4.6	14.3	2.3	2.2			6.7	9.7				1.4		12.2	4.2		*19.2*

Expected proportions of synonymous versus nonsynonymous changes, conditional on a base replacement mutation within a given amino acid site, are indicated in percentile. Blanks correspond to changes that require multiple mutational steps. An X designates termination codons. Expected proportions of synonymous changes are indicated in italics.

An obvious consequence of ENU mutagenesis is that because of the relatively low occurrence of G/C-to-C/G transversion changes (Table 1), some amino acid replacements would be disproportionately underrepresented (Table 2). As long as the expectation is based on the present collection that does not include any C-to-G transversions (see Table 1), it is suggested that seven of the amino acid changes should never be produced by ENU treatment (zero entries in Tables 2 and 3). Likewise, other seven amino acid replacements that are possible only by G-to-C transversions would be detected only in a rare occasion. While these two classes of mutation represent nearly 5% of disease-associated amino acid replacement changes observed in humans [22], they would comprise only 0.48% of the entire set of ENU-induced amino acid replacement changes. This imbalance suggests that a precise understanding of the mutational pattern and frequency of ENU-induced nucleotide changes would be desirable for properly designing and conducting a sequence-based mutagenesis experiment.

### Contrasting mutational patterns in phenotype- versus sequence-based screens

To see whether a similar pattern of biased mutation could also be detected in phenotype-based mutagenesis screens, we here contrast two classes of induced mutations, each derived from phenotype- and sequence-based screens, respectively. Previously, Justice et al. [1] provided a list of germline mutations detected in phenotype-based screens of ENU-mutagenized mice (see also [18]). Among 62 mutations recorded in Justice et al. [1], two mutations (*816SB *and *4494SB *at the *Myo7a *locus) accompany deletions [23], while other two (*b-m3H *and *b-m4H *at the *Gpi1 *locus) are considered to have originated from a single mutational event [24]. Hence 59 mutations are relevant for the present analysis. By reviewing the literature that are not included in Justice et al. [1], we have collected another 218 base replacement changes identified in phenotype-based screens, yielding a total of 277 ENU-induced germline mutations at 143 loci distributed across the 19 autosomes as well as the X chromosome in the mouse. The complete list of the relevant literature is provided as a supplementary material [see Additional file 1].

At first, it may appear that the overall pattern of germline mutations detected in phenotype-based screens is similar to the corresponding pattern in our sequence-based screen (Table 4); induced changes are found predominantly at T/A sites (75.1%). Moreover, whereas mutations at G/C sites show no clear sign of strand-specific effects (30 mutations at guanines versus 39 at cytosines on the nontranscribed strand), mutational changes at T/A sites occur more frequently when thymines and not adenines are located on the nontranscribed strand (136 versus 72). Whether the mutational pattern in phenotype-based screens should truly reflect the underlying distribution of ENU-induced mutations, however, still awaits further consideration.

**Table 4:** Reported number of base replacement changes in ENU mutagenesis

Base change		Phenotype- based screens		Sequence-based screens			
			
From	To			Germ cells ^a^		ES cells	
T	C	47	(17.0)	12	(14.5)	3	(9.7)
	A	76	(27.4)	15	(18.1)	4	(12.9)
	G	13	(4.7)	1	(1.2)	3	(9.7)
C	T	21	(7.6)	5	(6.0)	9	(29.0)
	A	16	(5.8)	4	(4.8)	1	(3.2)
	G	2	(0.7)	0	(0.0)	2	(6.5)
A	T	24	(8.7)	6	(7.2)	0	(0.0)
	C	7	(2.5)	1	(1.2)	0	(0.0)
	G	41	(14.8)	18	(21.7)	5	(16.1)
G	T	10	(3.6)	12	(14.5)	1	(3.2)
	C	0	(0.0)	1	(1.2)	0	(0.0)
	A	20	(7.2)	8	(9.6)	3	(9.7)

Total		277	(100.0)	83	(100.0)	31	(100.0)

Mutations are viewed as base changes on the nontranscribed strand. The relative proportion (in percentile) of each change is indicated in the parenthesis.

^a ^Germline mutations detected in Sakuraba et al. [13] are not included.

While it is expected that the mutational skew detected in our sequence-based screen (Table 1) is a faithful manifestation of the biased nature of ENU-induced germline mutation, the mutational pattern in phenotype-based screens (Table 4) may be confounded by several additional factors, including (i) unknown nucleotide composition of the genomic regions that are the potential target of the mutagenesis, and (ii) differential phenotypic effects caused by different base replacement mutations. These factors do not affect the mutational pattern in the sequence-based screens (although it may still be confounded due to nonrandom mutation discovery by the TGCE system, which we consider unlikely). For one reason, this is because the per-site mutation frequencies derived from the sequence-based analysis are, by definition, adjusted by the nucleotide composition of the targeted regions. Such adjustments are possible only when we base our analysis on appropriate sequence information, which is usually never obtained in the phenotype-based analysis. Moreover, while only those mutations that confer noticeable effects on the focal phenotype can be identified by phenotype-based screens, any mutational changes that fall within the targeted regions should in principle be identified by sequence-based screens, irrespective of their phenotypic effects. The former may hence represent a biased set of the latter, comprising a limited class of mutations that potentially has greater impacts on phenotypic function. Put differently, this implies that by investigating the incongruence between the two classes of mutations, we may be able to specify mutations with higher propensities of disrupting genome function.

As a possible sign of such incongruence, we here focus on a statistically significant difference detected for the patterns of mutational changes at thymines. In our sequence-based screen (Table 1), 57 mutations are found to alter thymines on the nontranscribed strand, among which 31 is replaced by cytosines, while 21 by adenines. Contrastingly, among 136 detected changes at thymines in the phenotype-based screens (Table 4), 76 are replaced by adenines, while only 47 result in replacement by cytosines (χ^2^_df = 1 _= 6.778, *P *= 0.009). Similar discrepancy between the two classes of mutations has been also pointed out by Augustin et al. [11]. Since T-to-A mutations may generate nonsense changes whereas T-to-C mutations do not, the excess of T-to-A mutations in phenotype-based screens may be because of the greater impact of nonsense changes on protein function. At first glance, this conjecture may appear valid, since nonsense changes are disproportionately overrepresented among mutations detected in phenotype-based screens (Table 5); among those mutations identified in the protein-coding sequences (i.e. synonymous and nonsynonymous mutations), only 4.6% (3/65) are nonsense in our sequence-based screen (in reasonable agreement with the theoretical expectation of 3.9%; see above), whereas in the phenotype-based screens, the corresponding proportion exceeds 20% (46 among 223 mutations). However, a closer look at the two classes of mutations unveils that the incongruence is also present even when only missense mutations are considered (Table 6). Since different subsets among the possible amino acid replacements are achieved by different base replacement changes, this observation suggests that amino acid replacements induced by T-to-A mutations are expected to affect protein function more radically on average, eventually leading to their excess representation in phenotype-based screens.

**Table 5:** Mutation types in ENU mutagenesis

	Phenotype-based screens	Sequence-based screens		
		
Mutation type		Present study	Germ cells ^a^	ES cells
5' flanking	0	1	1	0
Exonic				
5' untranslated region	2	0	1	0
Missense	175	44	45	18
Make-sense	2	1	1	0
Nonsense	46	3	4	0
Synonymous	0	17	15	9
3' untranslated region	0	1	2	0
Intronic ^b^	52	63	8	4
Not specified	0	0	6	0

Total	277	130	83	31

The number of occurrence for each type of mutation is listed.

^a ^Germline mutations detected in Sakuraba et al. [13] are not included.

^b ^Intronic mutations include base replacement changes leading to aberrant splicing.

**Table 6:** Mutation types in phenotype- versus sequence-based screens

	Phenotype-based screens		Sequence-based screen ^a^	
	
Mutation type	T-to-C	T-to-A	T-to-C	T-to-A
Missense	31	48	12	5
Make-sense	0	1	0	0
Nonsense	0	18	0	2
Synonymous	0	0	3	0
3' untranslated region	0	0	1	0
Intronic ^b^	16	9	15	14

Total	47	76	31	21

The number of T-to-C or T-to-A changes for each type of mutation is listed.

^a ^Only mutations detected in Sakuraba et al. [13] are included.

^b ^Intronic mutations include base replacement changes leading to aberrant splicing.

Tables 4 and 5 also summarize the patterns of ENU-induced germline mutations detected independently in other sequence-based mutagenesis screens [7-10,12,14], as well as of mutations detected in sequence-based screens of ENU-mutagenized ES cells [20,25]. (Mutations described in Augustin et al. [11] are not included because the base changes on the nontranscribed strand are not specified.) While the overall patterns may appear similar, they do not show a clear sign of mutational bias peculiar to our sequence-based screen (more T-to-C transition than T-to-A transversion mutations). Hence although we expect that different screening methods should identify distinct sets of induced changes, possibilities remain that other factors not discussed here further complicate the patterns of mutations detected in mutagenesis screens.

### Possible causes of the strand asymmetry

To account for the observed strand bias among mutations induced at T/A sites (Table 1), we here discuss the possibility that the mutational asymmetry is generated as a by-product of transcription-coupled repair (TCR) of DNA damages. The process of TCR has been considered as a candidate responsible for the compositional strand asymmetries in the transcribed portion of the mammalian genome [26-32].

Transcription overexposes the nontranscribed strand to DNA damage, thereby biasing the occurrence of mutations between the two strands. Based on comparative analyses of orthologous sequences, it has recently been hypothesized that the compositional asymmetries in mammalian transcribed regions are indeed caused by the mutational bias inherent in TCR [26]. Despite some criticisms (e.g. [33]), the hypothesis has gained a further support from comprehensive analyses of human gene expression, which have detected a significant and positive correlation between the extent of compositional asymmetry and the expression level in the germline [28,31,32].

If TCR indeed plays a major part in generating the compositional strand asymmetries in the mammalian genome, then it should also affect the strand-specific pattern of experimentally induced mutation in a predictable manner. As reported previously [4,5], ENU introduces heritable sequence changes predominantly at T/A sites by adding *O*^2^- or *O*^4^-ethyl adducts on thymines. Consequently, if transcription-associated DNA repair mechanisms (such as TCR) should effectively remove molecular lesions on the transcribed but not on the complementary nontranscribed strand of DNA, the resulting pattern of mutations on the nontranscribed strand should be strongly skewed toward changes at thymines; adenine modifications, on the other hand, should be less frequently observed.

Based on a genome-wide set of heritable mutations obtained from our sequence-based screen, the present study clearly demonstrates that ENU-induced changes in the mouse germline indeed follow the predicted pattern (Table 1). Although similar observations have been made for induced changes at the *Hprt *locus [34-36], previous reports are based on somatic mutations isolated from phenotypic assays using 6-thioguanine as the selective chemical agent; namely, the mutations are not heritable and the screening methods are not sequence-based. Estimated mutation frequencies are not adjusted by the base composition of the targeted regions either. Therefore, possibilities cannot be excluded that the observed mutational pattern may have been confounded by the region-specific nucleotide composition, and also by the phenotypic effects of induced changes. As detailed above, these factors are adequately controlled in the present analysis.

While premutagenic lesions including *O*^6^- and *N*^7^-ethylguanines are formed at even higher rates *in vivo *by ENU [37], these DNA damages are repaired rather rapidly by mechanisms other than TCR [4,5], eventually making only a minor contribution to heritable changes. The absence of mutational bias at G/C sites (Table 1) may further lend support for the view that TCR, and not the other transcription-associated mutagenic mechanisms such as cytosine deamination [38,39], is the primary cause of compositional asymmetries in the mammalian transcribed regions [26]. Moreover, since some forms of DNA repair eliminate lesions by preferentially killing the damaged cells [40], the selective removal of the lesions on the transcribed strand may be promoted by the apoptotic effect of strand-specific repair mechanisms.

The strand-specific effects of TCR should be observed among heritable mutations only when mutated nucleotide sites are transcribed in the germline. In other words, strand asymmetry would not be observed when mutations are induced in the nontranscribed portion of the genome, which might include intergenic sequences and pseudogenes. (A caveat here is that some pseudogene sequences retain transcriptional activity while loosing their primary ability of encoding amino acid sequences [41-44].) Alternatively, it is also expected that the strand-specific mutational pattern should be absent in genic regions if they are coupled with antisense transcripts [45,46]. When genes and their antisense products are coexpressed [47], transcription-associated effects should influence both sense and antisense strands equally, thereby erasing the vestiges of strand-specific effects.

Although we have not experimentally confirmed the transcription status in the mouse germ cells of the 54 genic regions screened in our analysis, available data retrieved from the mouse Gene Expression Database (GXD) [48] suggest that in testis, 17 regions are transcribed, whereas none has been demonstrated to be nontranscribed; for the remaining 37 regions, no appropriate information currently exists. The genomic regions included in our sequence-based screen are chosen without any prior knowledge for their function or expression status in the germline. Recalling that some level of germline transcription would involve a large fraction of human genes (71 – 91% [28]), we expect that most of the remaining 37 sequences are also transcribed in the mouse germ cells, even if they may not be essential for germline function. By contrasting the patterns of experimentally induced germline mutations in genomic regions with distinct transcriptional properties, we would gain a more clarified view for the role of transcription in generating mutational and eventual compositional strand asymmetries.

## Conclusion

Based on a detailed evaluation of the screening data obtained from our large-scale mutagenesis experiment, the present analysis clearly illustrates the biased nature of ENU-induced mutations, and discusses the possible causes and consequences of the nonuniformity. Despite the mutational bias inherent in ENU mutagenesis, however, this study also provides a strong support for its utility in obtaining a series of allelic variants at a genetic locus. We expect that the present findings will be useful in promoting the efficient production of mutant lines harboring amino acid variants. More generally, by enhancing the collection of experimentally induced mutations in unambiguously defined genomic regions, sequence-based mutagenesis studies will further illuminate the molecular basis of mutagenic and repair mechanisms that preferentially produce a certain class of mutational changes over others.

## Methods

### Sequence-based mutagenesis screen

The detailed method of our sequence-based mutagenesis is documented in Sakuraba et al. [13]. In brief, male C57BL/6J mice were injected intraperitoneally with an ENU dose of 85 or 100 mg/kg at 8–10 weeks of age. The injections were carried out twice at weekly intervals. The ENU-treated males were then mated to DBA/2J or C3H/HeJ females to obtain G1 offspring, which should harbor ENU-induced mutations as heterozygotes. We have chosen 63 genomic regions (54 genic and nine putatively intergenic) for the target-selected mutagenesis and designed 199 primer pairs therein (see Table 1 in [13]). Most of the primer pairs targeted for the genic sequences were designed to cover exonic regions, which were aimed at detecting induced mutations leading to amino acid sequence alterations, while only a few extended to nontranscribed flanking sequences. Heritable changes in the G1 males were screened using a temperature gradient capillary electrophoresis (TGCE) system, which eventually summed up to a screen of nearly 2 × 10^8 ^nucleotide sites in total [13].

Since we are interested in obtaining the strand-specific pattern of induced changes, we here concentrate on mutations identified in the 54 genic regions, where the two strands of the genomic DNA can uniquely be discriminated (namely, sense versus antisense strands). By targeting more than 50 loci that together embrace all the 19 autosomes in the mouse, the present analysis should be free from locus- or region-specific effects that may obscure the genuine pattern of ENU-induced mutation. The GC content of the whole screened sites is 48.0% (see also Table 1), which is somewhat lower than the averages for the mouse exonic sequences (52.0 – 57.1% [49]), but well above the genome-wide average of 42% in mice [50].

The detected mutations are classified according to nucleotide changes on the nontranscribed (or sense) strand. Complementary mutations (e.g. A-to-T versus T-to-A changes) are therefore distinguished and considered different from each other. Note that this distinction cannot be made for sequence changes detected in nongenic regions. The rare occasions of mutations producing length variants (insertions and deletions) are also excluded from the analysis.

### Per-site frequency of induced mutations

Given the observed number *k*_*ij *_for mutations from nucleotide *i *to *j*, (*i*, *j *∈ {T, C, A, G}, *j *≠ *i*), we compute the mutation frequency per nucleotide site as μ_*ij *_= *k*_*ij*_/*L*_*i*_, where *L*_*i *_represents the total count of nucleotide *i *within the genomic region covered in the screen. The average frequency of mutation at nucleotide *i *is simply given by μ_*i *_= Σ_*j*_μ_*ij *_= Σ_*j*_*k*_*ij*_/*L*_*i*_. Likewise, the overall frequency of mutation per nucleotide site is obtained as Σ_*ij*_*k*_*ij*_/Σ_*i*_*L*_*i*_.

Assuming that the probability of observing *k *or fewer mutations within *L *screened sites should follow a poisson distribution *P*{*X *≤ *k*} = Σ^*k*^_κ = 0 _[*e*^-*L*μ ^(*L*μ)^κ ^/κ!] (where κ designates a dummy variable), we determine the 95% confidence interval (CI) for the mutation frequency μ by solving this equation with *P *= 0.025 and 0.975. When no mutations were observed (as for the C-to-G transversion in our collection; see Table 1), we solved the equation *P*{*X *= 0} = *e*^-*L*μ ^= 0.05 to obtain an upper limit for the mutation frequency.

### Expected pattern of amino acid changes

To infer the effect of ENU mutagenesis on the mouse protein sequences, the expected pattern of amino acid replacement changes is computed by superimposing the distribution of ENU-induced base replacement mutations onto the pattern of codon usage in the mouse coding sequences. For each of the 64 triplet codons, there are nine codons that could be reached by single base replacement. The relative frequency distribution of 576 (= 64 × 9) possible codon replacements is obtained simply by multiplying the abundance of the original codon in the mouse coding sequences, as described in the Codon Usage Database [21] based on GenBank Release 151.0 (19 December, 2005), by the per-site frequency of appropriate base replacement mutation (as derived above). Base replacement mutation in a codon is either synonymous or nonsynonymous; the latter leads to an amino acid sequence alteration, while the former preserves the coded amino acid despite the change in the DNA sequence. (Mutation that preserves a termination codon (e.g. TAA-to-TAG) is here considered as synonymous.) Nonsynonymous mutation is further subdivided into missense (exchange of an amino acid by another amino acid), nonsense (replacement of an amino acid by a termination codon), or make-sense (replacement of a termination codon by one of the 20 amino acids) mutation. The expected distribution of amino acid replacement changes is obtained from the derived pattern of codon replacement, by summing up the appropriate codons in case of degeneracy. The result is summarized in Table 2.

### Literature survey

We carried out a comprehensive literature survey and collected reported instances of ENU-induced base replacement mutations detected in phenotype-based mutagenesis screens. This has been done by adding germline mutations that are not included in the previous list [1,18]. We do not claim to have included all the relevant studies, but we expect that the collection should represent an unbiased sample of the literature. An additional literature survey has been conducted also for germline mutations detected independently in other sequence-based studies, as well as for mutations identified in mutagenized embryonic stem (ES) cell systems.

## Authors' contributions

KRT and YS summarized the screening data. KRT conducted the data analysis and wrote the paper. YS performed the mutation screening. YG led the entire project. All authors read and approved the final manuscript.

## Supplementary Material

Additional File 1 List of publications reporting base replacement mutations detected in phenotype-based mutagenesis screens. This pdf file contains a complete list of published research articles that document ENU-induced base replacement changes detected in phenotype-based mutagenesis screens. Click here for file
