# Supplementary material for: Mutational pattern and frequency of induced nucleotide changes in mouse ENU mutagenesis
Source: BMC Mol Biol. 2007 Jun 20;8:52. doi: 10.1186/1471-2199-8-52 (PMC1914352; doi:10.1186/1471-2199-8-52)
Supplement: Additional File 1 — List of publications reporting base replacement mutations detected in phenotype-based mutagenesis screens. This pdf file contains a complete list of published research articles that document ENU-induced base replacement changes detected in phenotype-based mutagenesis screens. [file 1471-2199-8-52-S1.pdf]

# Mutational pattern and frequency of induced nucleotide changes in mouse ENU mutagenesis

K Ryo Takahasi, Yoshiyuki Sakuraba and Yoichi Gondo

## List of publications reporting base replacement mutations detected in phenotype-based mutagenesis screens

Our comprehensive literature survey has found a total of 277 instances of ENU-induced base replacement changes detected in phenotype-based mutagenesis screens. They are documented in the following 161 research articles.

1. Ackerman, K. G., B. J. Herron, S. O. Vargas, H. Huang, S. G. Tevosian *et al.*, 2005 *Fog2* is required for normal diaphragm and lung development in mice and humans. PLoS Genet. 1: e10.

2. Ahituv, N., A. Erven, H. Fuchs, K. Guy, R. Ashery-Padan *et al.*, 2004 An ENU-induced mutation in AP-2α leads to middle ear and ocular defects in Doarad mice. Mamm. Genome 15: 424-432.

3. Alexander, W. S., E. M. Viney, J.-G. Zhang, D. Metcalf, M. Kauppi *et al.*, 2006 Thrombocytopenia and kidney disease in mice with a mutation in the *C1galt1* gene. Proc. Natl. Acad. Sci. USA 103: 16442-16447.

4. Aponte, J. L., G. A. Sega, L. J. Hauser, M. S. Dhar, C. M. Withrow *et al.*, 2001 Point mutations in the murine fumarylacetoacetate hydrolase gene: animal models for the human genetic disorder hereditary tyrosinemia type 1. Proc. Natl. Acad. Sci. USA 98: 641-645.

5. Besson, V., V. Nalesso, A. Herpin, J.-C. Bizot, N. Messaddeq *et al.*, 2005 Training and aging modulate the loss-of-balance phenotype observed in a new ENU-induced allele of *Otopetrin1*. Biol. Cell 97: 787-798.

6. Bogani, D., N. Warr, P. Elms, J. Davies, Z. Tymowska-Lalanne *et al.*, 2004 New semidominant mutations that affect mouse development. genesis 40: 109-117.

7. Bosman, E. A., A. C. Penn, J. C. Ambrose, R. Kettleborough, D. L. Stemple *et al.*, 2005 Multiple mutations in mouse *Chd7* provide models for CHARGE syndrome. Hum. Mol. Genet. 14: 3463-3476.

8. Boulechfar, S., J. Lamoril, X. Montagutelli, J. L. Guenet, J. C. Deybach *et al.*, 1993 Ferrochelatase structural mutant ($Fech^{m1Pas}$) in the house mouse. Genomics 16: 645-648.

9. Brannan, C. I., M. A. Bedell, J. L. Resnick, J. J. Eppig, M. A. Handel *et al.*, 1992 Developmental abnormalities in $Steel^{17H}$ mice result from a splicing defect in the steel factor cytoplasmic tail. Genes Dev. 6: 1832-1842.

10. Buchner, D. A., K. L. Seburn, W. N. Frankel and M. H. Meisler, 2004 Three ENU-induced neurological mutations in the pore loop of sodium channel *Scn8a* ($Na_v1.6$) and a genetically linked retinal mutation, *rd13*. Mamm. Genome 15: 344-351.

11. Bultman, S. J., T. C. Gebuhr and T. Magnuson, 2005 A Brg1 mutation that uncouples ATPase activity from chromatin remodeling reveals an essential role for SWI/SNF-related complexes in β-globin expression and erythroid development. Genes Dev. 19: 2849-2861.

12. Carpinelli, M. R., I. P. Wicks, N. A. Sims, K. O'Donnell, K. Hanzinikolas *et al.*, 2002 An ethyl-nitrosourea-induced point mutation in *Phex* causes exon skipping, X-linked hypophosphatemia, and rickets. Am. J. Pathol. 161: 1925-1933.

13. Carpinelli, M. R., D. J. Hilton, D. Metcalf, J. L. Antonchuk, C. D. Hyland *et al.*, 2004 Suppressor screen in *Mpl*$^{-/-}$ mice: *c-Myb* mutation causes supraphysiological production of platelets in the absence of thrombopoietin signaling. Proc. Natl. Acad. Sci. USA 101: 6553-6558.

14. Caspary, T., M. J. García-García, D. Huangfu, J. T. Effenschwiler, M. R. Wyler *et al.*, 2002 Mouse *Dispatched homolog1* is required for long-range, but not juxtacrine, Hh signaling. Curr. Biol. 12: 1628-1632.

15. Clapcott, S. J., J. Peters, P. C. Orban, R. Brambilla and C. F. Graham, 2003 Two ENU-induced mutations in *Rasgrf1* and early mouse growth retardation. Mamm. Genome 14: 495-505.

16. Cordes, S. P. and G. S. Barsh, 1994 The mouse segmentation gene *kr* encodes a novel basic domain-leucine zipper transcription factor. Cell 79: 1025-1034.

17. Cox, G. A., S. F. Phelps, V. M. Chapman and J. S. Chamberlain, 1993 New mdx mutation disrupts expression of muscle and nonmuscle isoforms of dystrophin. Nat. Genet. 4: 87-93.

18. Cox, R. D., A. Hugill, A. Shedlovsky, J. K. Noveroske, S. Best *et al.*, 1999 Contrasting effects of ENU-induced embryonic lethal mutations of the *quaking* gene. Genomics 57: 333-341.

19. Cross, S. H., J. E. Morgan, A. Pattyn, K. West, L. McKie *et al.*, 2004 Haploinsufficiency for *Phox2b* in mice causes dilated pupils and atrophy of the ciliary ganglion: mechanistic insights into human congenital central hypoventilation syndrome. Hum. Mol. Genet. 13: 1433-1439.

20. Crozat, K., P. Georgel, S. Rutschmann, N. Mann, X. Du *et al.*, 2006 Analysis of the MCMV resistome by ENU mutagenesis. Mamm. Genome 17: 398-406.

21. Culiat, C. T., M. L. Klebig, Z. Liu, H. Monroe, B. Stanford *et al.*, 2005 Identification of mutations from phenotype-driven ENU mutagenesis in mouse Chromosome 7. Mamm. Genome 16: 555-566.

22. Curtin, J. A., E. Quint, V. Tsipouri, R. M. Arkell, B. Cattanach *et al.*, 2003 Mutation of *Celsr1* disrupts planar polarity of inner ear hair cells and causes severe neural tube defects in the mouse. Curr. Biol. 13: 1129-1133.

23. Desai, J., M. E. Shannon, M. D. Johnson, D. W. Ruff, L. A. Hughes *et al.*, 2006 *Nell1*-deficient mice have reduced expression of extracellular matrix proteins causing cranial and vertebral defects. Hum. Mol. Genet. 15: 1329-1341.

24. Du, X., K. Tabeta, K. Hoebe, H. Liu, N. Mann *et al.*, 2004 *Velvet*, a dominant *Egfr* mutation that causes wavy hair and defective eyelid development in mice. Genetics 166: 331-340.

25. Du, X., K. Tabeta, N. Mann, K. Crozat, S. Mudd *et al.*, 2005 An essential role for *Rxr*α in the development of Th2 responses. Eur. J. Immunol. 35: 3414-3423.

26. Eggenschwiler, J. T., E. Espinoza and K. V. Anderson, 2001 Rab23 is an essential negative regulator of the mouse Sonic hedgehog signalling pathway. Nature 412: 194-198.

27. Elms, P., P. Siggers, D. Napper, A. Greenfield and R. Arkell, 2003 *Zic2* is required for neural crest formation and hindbrain patterning during mouse development. Dev. Biol. 264: 391-406.

28. Favor, J., H. Peters, T. Hermann, W. Schmahl, B. Chatterjee *et al.*, 2001 Molecular characterization of $Pax6^{2Neu}$ through $Pax6^{10Neu}$: an extension of the Pax6 allelic series and the identification of two possible hypomorph alleles in the mouse *Mus musculus*. Genetics 159: 2689-1700.

29. Fernández-Valdivia, R., Y. Zhang, S. Pai, M. L. Metxker and A. Schumacher, 2006 *l7Rn6* encodes a novel protein required for Clara cell function in mouse lung development. Genetics 172: 389-399.

30. Fitch, K. R., K. A. McGowan, C. D. van Raamsdonk, H. Fuchs, D. Lee *et al.*, 2003 Genetics of dark skin in mice. Genes Dev. 17: 214-228.

31. Flenniken, A. M., L. R. Osborne, N. Anderson, N. Ciliberti, C. Fleming *et al.*, 2005 A *Gja1* missense mutation in a mouse model of oculodentodigital dysplasia. Development 132: 4375-4386.

32. García-García, M. J. and K. V. Anderson, 2003 Essential role of glycosaminoglycans in Fgf signaling during mouse gastrulation. Cell 114: 727-737.

33. García-García, M. J., J. T. Eggenschwiler, T. Caspary, H. L. Alcorn, M. R. Wyler *et al.*, 2005 Analysis of mouse embryonic patterning and morphogenesis by forward genetics. Proc. Natl. Acad. Sci. USA 102: 5913-5919.

34. García-Martínez, L. F., M. W. Appleby, K. Staehling-hampton, D. M. Andrews, Y. Chen *et al.*, 2004 A novel mutation in CD83 results in the development of a unique population of $CD4^+$ T cells. J. Immunol. 173: 2995-3001.

35. Georgel, P., K. Crozat, X. Lauth, E. Makrantonaki, H. Seltmann *et al.*, 2005 A Toll-like receptor 2-responsive lipid effector pathway protects mammals against skin infections with gram-positive bacteria. Infect. Immun. 73: 4512-4521.

36. Graw, J., M. Jung, J. Löster, N. Klopp, D. Soewarto *et al.*, 1999 Mutation in the βA3/A1-crystallin encoding gene *Cryba1* causes a dominant cataract in the mouse. Genomics 62: 67-73.

37. Graw, J., N. Klopp, J. Löster, D. Soewarto, H. Fuchs *et al.*, 2001 Ethylnitrosourea-induced mutation in mice leads to the expression of a novel protein in the eye and to dominant cataracts. Genetics 157: 1313-1320.

38. Graw, J., J. Löster, D. Soewarto, H. Fuchs, B. Meyer *et al.*, 2001 Characterization of a mutation in the lens-specific MP70 encoding gene of the mouse leading to a dominant cataract. Exp. Eye Res. 73: 867-876.

39. Graw, J., J. Löster, D. Soewarto, H. Fuchs, A. Reis *et al.*, 2001 *Aey2*, a new mutation in the βB2-crystallin-encoding gene of the mouse. Invest. Ophthalmol. Vis. Sci. 42: 1574-1580.

40. Graw, J., J. Löster, D. Soewarto, H. Fuchs, B. Meyer *et al.*, 2001 Characterization of a new, dominant V124E mutation in the mouse αA-crystallin-encoding gene. Invest. Ophthalmol. Vis. Sci. 42: 2909-2915.

41. Graw, J., J. Löster, D. Soewarto, H. Fuchs, A. Reis *et al.*, 2002 V76D mutation in a conserved γD-crystallin region leads to dominant cataracts in mice. Mamm. Genome 13: 452-455.

42. Graw, J., A. Neuhäuser-Klaus, J. Löster, N. Klopp and J. Favor, 2002 Ethylnitrosourea-induced base pair substitution affects splicing of the mouse γE-crystallin encoding gene leading to the expression of a hybrid protein and to a cataract. Genetics 161: 1633-1640.

43. Graw, J., A. Neuhäuser-Klaus, N. Klopp, P. B. Selby, J. Löster *et al.*, 2004 Genetic and allelic heterogeneity of *Cryg* mutations in eight distinct forms of dominant cataract in the mouse. Invest. Ophthalmol. Vis. Sci. 45: 1202-1213.

44. Graw, J., J. Löster, O. Puk, D. Münster, N. Haubst *et al.*, 2005 Three novel *Pax6* alleles in the mouse leading to the same small-eye phenotype caused by different consequences at target promoters. Invest. Ophthalmol. Vis. Sci. 46: 4671-4683.

45. Gronemeier, M., A. Condie, J. Prosser, K. Steinmeyer, T. J. Jentsch *et al.*, 1994 Nonsense and missense mutations in the muscular chloride channel gene *Clc-1* of myotonic mice. J. Biol. Chem. 269: 5963-5967.

46. Grosse, J., V. Chitu, A. Marquardt, P. Hanke, C. Schmittwolf *et al.*, 2006 Mutation of mouse *Mayp*/*Pstpip2* causes a macrophage autoinflammatory disease. Blood 107: 3350-3358.

47. Haefele, M. J., G. White and J. D. McDonald, 2001 Characterization of the mouse phenylalanine hydroxylase mutation $Pah^{enu3}$. Mol. Genet. Metab. 72: 27-30.

48. Hafezparast, M., R. Klocke, C. Ruhrberg, A. Marquardt, A. Ahmad-Annuar *et al.*, 2003 Mutations in dynein link motor neuron degeneration to defects in retrograde transport. Science 300: 808-812.

49. Hallsson, J. H., J. Favor, C. Hodgkinson, T. Glaser, M. L. Lamoreux *et al.*, 2000 Genomic, transcriptional and mutational analysis of the mouse *microphthalmia* locus. Genetics 155: 291-300.

50. Hansdottir, A. G., K. Pálsdóttir, J. Favor, A. Neuhäuser-Klaus, H. Fuchs *et al.*, 2004 The novel mouse microphthalmia mutations *Mitf*$^{mi-enu5}$ and *Mitf*$^{mi-bcc2}$ produce dominant negative Mitf proteins. Genomics 83: 932-935.

51. Hart, A. W., L. McKie, J. E. Morgan, P. Gautier, K. West *et al.*, 2005 Genotype-phenotype correlation of mouse *Pde6b* mutations. Invest. Ophthalmol. Vis. Sci. 46: 3443-3450.

52. Hart, A. W., J. E. Morgan, J. Schneider, K. West, L. McKie *et al.*, 2006 Cardiac malformations and midline skeletal defects in mice lacking filamin A. Hum. Mol. Genet. 15: 2457-2467.

53. Hentges, K. E., B. Sirry A.-C. Gingeras, D. Sarbassov, N. Sonenberg *et al.*, 2002 FRAP/mTOR is required for proliferation and patterning during embryonic development in the mouse. Proc. Natl. Acad. Sci. USA 98: 13796-13801.

54. Herron, B. J., W. Lu, C. Rao, S. Liu, H. Peters *et al.*, 2002 Efficient generation and mapping of recessive developmental mutations using ENU mutagenesis. Nat. Genet. 30: 185-189.

55. Hill, R. E., J. Favor, B. L. M. Hogan, C. C. T. Ton, G. F. Saunders *et al.*, 1991 Mouse *Small* eye results from mutations in a paired-like homeobox-containing gene. Nature 354: 522-525.

56. Hoebe, K., P. Georgel, S. Rutschmann, X. Du, S. Mudd *et al.*, 2005 CD36 is a sensor of diacylglycerides. Nature 433: 523-527.

57. Hong, H.-K., J. K. Noveroske, D. J. Headon, T. Liu, M.-S. Sy *et al.*, 2001 The winged helix/forkhead transcription factor *Foxq1* regulates differentiation of hair in satin mice. genesis 29: 163-171.

58. Huang, J.-D., M. Jamie, T. V. Cope, V. Mermall, M. C. Strobel *et al.*, 1998 Molecular genetic dissection of mouse unconventional myosin-VA: head region mutations. Genetics 148: 1951-1961.

59. Huang, J.-D., V. Mermall, M. C. Strobel, L. B. Russell, M. S. Mooseker *et al.*, 1998 Molecular genetic dissection of mouse unconventional myosin-VA: tail region mutations. Genetics 148: 1963-1972.

60. Huangfu, D., and K. V. Anderson, 2005 Cilia and Hedgehog responsiveness in the mouse. Proc. Natl. Acad. Sci. USA 102:11325-11330.

61. Huangfu, D., A. Liu, A. S. Rakeman, N. S. Murcia, L. Niswander *et al.*, 2003 Hedgehog signalling in the mouse requires intraflagellar transport proteins. Nature 426: 83-87.

62. Hurle, B., E. Ignatova, S. M. Massironi, T. Mashimo, X. Rios *et al.*, 2003 Non-syndromic vestibular disorder with otoconial agenesis in *tilted/mergulhador* mice caused by mutations in otopetrin 1. Hum. Mol. Genet. 12: 777-789.

63. Hustad, C. M., W. L. Perry, L. D. Siracusa, C. Rasberry, L. Cobb *et al.*, 1995 Molecular genetic characterization of six recessive viable alleles of the mouse *agouti* locus. Genetics 140: 255-265.

64. Im, W. B., S. F. Phelps, E. H. Copen, E. G. Adams, J. L. Slightom *et al.*, 1996 Differential expression of dystrophin isoforms in strains of *mdx* mice with different mutations. Hum. Mol. Genet. 5: 1149-1153.

65. Inoue, M., Y. Sakuraba, H. Motegi, N. Kubota, H. Toki *et al.*, 2004 A series of maturity onset diabetes of the young, type 2 (MODY2) mouse models generated by a large-scale ENU mutagenesis program. Hum. Mol. Genet. 13: 1147-1157.

66. Isaacs, A. M., K. E. Davies, A. J. Hunter, P. M. Nolan, L. Vizor *et al.*, 2000 Identification of two new *Pmp22* mouse mutants using large-scale mutagenesis and a novel rapid mapping strategy. Hum. Mol. Genet. 9: 1865-1871.

67. Isaacs, A. M., A. Jeans, P. L. Oliver, L. Vizor, S. D. M. Brown *et al.*, 2002 Identification of a new *Pmp22* mouse mutant and trafficking analysis of a *Pmp22* allelic series suggesting that protein aggregates may be protective in *Pmp22*-associated peripheral neuropathy. Mol. Cell. Neurosci. 21: 114-125.

68. Isaacs, A. M., P. L. Oliver, E. L. Jones, A. Jeans, A. Potter *et al.*, 2003 A mutation in *Af4* is predicted to cause cerebellar ataxia and cataracts in the robotic mouse. J. Neurosci. 23: 1631-1637.

69. Jablonski, M. M., C. Dalke, X. Wang, L. Lu, K. F. Manly *et al.*, 2005 An ENU-induced mutation in *Rs1h* causes disruption of retinal structure and function. Mol. Vis. 11: 569-581.

70. Jaubert, J., F. Jaubert, N. Martin, L. L. Washburn, B. K. Lee *et al.*, 1999 Three new allelic mouse mutations that cause skeletal overgrowth involve the natriuretic peptide receptor C gene (*Npr3*). Proc. Natl. Acad. Sci. USA 96: 10278-10283.

71. Jean, J. C., C. O. Harding, S. M. Oakes, Q. Yu, P. K. Held *et al.*, 1999 γ-Glutamyl transferase (GGT) deficiency in the $GGT^{enu1}$ mouse results from a single point mutation that leads to a stop codon in the first coding exon of GGT mRNA. Mutagenesis 14: 31-36.

72. Ji, Y., M. J. Walkowicz, K. Buiting, D. K. Johnson, R. E. Tarvin *et al.*, 1999 The ancestral gene for transcribed, low-copy repeats in the Prader-Willi/Angelman region encodes a large protein implicated in protein trafficking, which is deficient in mice with neuromuscular and spermiogenic abnormalities. Hum. Mol. Genet. 8: 533-542.

73. Jiang, Z., P. Georgel, X. Du, L. Shamel, S. Sovath *et al.*, 2005 CD14 is required for MyD88-independent LPS signaling. Nat. Immunol. 6: 565-570.

74. Jiang, Z., P. Georgel, C. Li, J. Choe, K. Crozat *et al.*, 2006 Details of Toll-like receptor:adapter interaction revealed by germ-line mutagenesis. Proc. Natl. Acad. Sci. USA 103: 10961-10966.

75. Jun, J. E., L. E. Wilson, C. G. Vinuesa, S. Lesage, M. Blery *et al.*, 2003 Identifying the MAGUK protein Carma-1 as a central regulator of humoral immune responses and atopy by genome-wide mouse mutagenesis. Immunity 18: 751-762.

76. Kapfhamer, D., O. Valladares, Y. Sun, P. M. Nolan, J. J. Rux *et al.*, 2002 Mutations in *Rab3a* alter circadian period and homeostatic response to sleep loss in the mouse. Nat. Genet. 32: 290-295.

77. Karkkainen, M. J., A. Saaristo, L. Jussila, L. A. Karila, E. C. Lawrence *et al.*, 2001 A model for gene therapy of human hereditary lymphedema. Proc. Natl. Acad. Sci. USA 98: 12677-12682.

78. Kearney, J. A., Y. Yang, B. Beyer, S. K. Bergren, L. Claes *et al.*, 2006 Severe epilepsy resulting from genetic interaction between *Scn2a* and *Kcnq2*. Hum. Mol. Genet. 15: 1043-1048.

79. Kibar, Z., K. J. Vogan, N. Groulx, M. J. Justice, D. A. Underhill *et al.*, 2001 *Ltap*, a mammalian homolog of *Drosophila Strabismus/Van Gogh*, is altered in the mouse neural tube mutant Loop-tail. Nat. Genet. 28: 251-255.

80. Kiernan, A. E., N. Ahituv, H. Fuchs, R. Balling, K. B. Avraham *et al.*, 2001 The Notch ligand *Jagged1* is required for inner ear sensory development. Proc. Natl. Acad. Sci. USA 98: 3873-3878.

81. Kikkawa, Y., A. Oyama, R. Ishii, I. Miura, T. Amano *et al.*, 2003 A small deletion hotspot in the type II keratin gene *mK6irs1/Krt2-6g* on mouse chromosome 15, a candidate for causing the wavy hair of the caracul (*Ca*) mutation. Genetics 165: 721-733.

82. Kile, B. T., K. E. Hentges, A. T. Clark, H. Nakamura, A. P. Salinger *et al.*, 2003 Functional genetic analysis of mouse chromosome 11. Nature 425: 81-86.

83. King, D. P., Y. Zhao, A. M. Sangoram, L. D. Wilsbacher, M. Tanaka *et al.*, 1997 Positional cloning of the mouse circadian *Clock* gene. Cell 89: 641-653.

84. Klebig, M. L., M. D. Wall, M. D. Potter, E. L. Rowe, D. A. Carpenter *et al.*, 2003 Mutations in the clathrin-assembly gene *Picalm* responsible for the hematopoietic and iron metabolism abnormalities in *fit1* mice. Proc. Natl. Acad. Sci. USA 100: 8360-8365.

85. Kljuic, A., H. Bazzi, J. P. Sundberg, A. Martinez-Mir, R. O'Shaunghnessy *et al.*, 2003 Desmoglein 4 in hair follicle differentiation and epidermal adhesion: evidence from inherited hypotrichosis and acquired pemphigus vulgaris. Cell 113: 249-260.

86. Klopp, N., J. Favor, J. Löster, R. B. Lutz, A. Neuhäuser-Klaus *et al.*, 1998 Three murine cataract mutants (*Cat2*) are defective in different γ-crystallin genes. Genomics 52: 152-158.

87. Lee, D., S. H. Cross, K. E. Strunk, J. E. Morgan, C. L. Bailey *et al.*, 2004 *Wa5* is a novel ENU-induced antimorphic allele of the epidermal growth factor receptor. Mamm. Genome. 15: 525-536.

88. Lewis, S. E., F. M. Johnson, L. C. Skow, D. Popp, L. B. Barnett *et al.*, 1985 A mutation in the β-globin gene detected in the progeny of a female mouse treated with ethylnitrosourea. Proc. Natl. Acad. Sci. USA 82: 5829-5831.

89. Liu, H., X. Du, M. Wang, Q. Huang, L. Ding *et al.*, 2005 Crystallin γB-I4F mutant protein binds to α-crystallin and affects lens transparency. J. Biol. Chem. 280: 25071-25078.

90. Lloyd, D. J., F. W. Hall, L. M. Tarantino and N. Gekakis, 2005 Diabetes insipidus in mice with a mutation in aquaporin-2. PLoS Genet. 1: e20.

91. Lloyd, D. J., S. Bohan and N. Gekakis, 2006 Obesity, hyperphagia and increased metabolic efficiency in Pc1 mutant mice. Hum. Mol. Genet. 15: 1884-1893.

92. Lucas, M. E., Q. Ma, D. Cunningham, J. Peters, B. Cattanach *et al.*, 2005 Identification of two novel mutations in the murine *Nsdhl* sterol dehydrogenase gene and development of a functional complementation assay in yeast. Mol. Genet. Metab. 80: 227-233.

93. Lyon, M. F., D. Bogani, Y. Boyd, P. Guillot and J. Favor, 2000 Further genetic analysis of two autosomal dominant mouse eye defects, *Ccw* and $Pax6^{coop}$. Mol. Vis. 6: 199-203.

94. Majewski, I. J., D. Metcalf, L. A. Mielke, D. L. Krebs, S. Ellis *et al.*, 2006 A mutation in the translation initiation codon of *Gata-1* disrupts megakaryocyte maturation and causes thrombocytopenia. Proc. Natl. Acad. Sci. USA 103: 14146-14151.

95. Marker, P. C., K. Seung, A. E. Bland, L. B. Russell and D. M. Kingsley, 1997 Spectrum of *Bmp5* mutations from germline mutagenesis experiments in mice. Genetics 145: 435-443.

96. Masuya, H., K. Shimizu, H. Sezutsu, Y. Sakuraba, J. Nagano *et al.*, 2005 Enamelin (*Enam*) is essential for amelogenesis: ENU-induced mouse mutants as models for different clinical subtypes of human amelogenesis imperfecta (AI). Hum. Mol. Genet. 14: 575-583.

97. Mburu, P., X. Z. Liu, J. Walsh, D. Saw, Jr., M. J. T. V. Cope *et al.*, 1997 Mutation analysis of the mouse myosin VIIA deafness gene. Genes Funct. 1: 191-203.

98. McDonald, J. D. and C. K. Charlton, 1997 Characterization of mutations at the mouse phenylalanine hydroxylase locus. Genomics 39: 402-405.

99. McGowan, K. A., S. Aradhya, H. Fuchs, M. H. de Angelis and G. S. Barsh, 2006 A mouse *Keratin 1* mutation causes dark skin and epidermolytic hyperkeratosis. J. Invest. Dermatol. 126: 1013-1016.

100. Meyer, C. W. E., D. Korthaus, W. Jagla, E. Cornali, J. Grosse *et al.*, 2004 A novel missense mutation in the mouse growth hormone gene causes semidominant dwarfism, hyperghrelinemia and obesity. Endocrinology 145: 2531-2541.

101. Miltenberger, R. J., K. Wakamatsu, S. Ito, R. P. Woychik, L. B. Russell *et al.*, 2002 Molecular and phenotypic analysis of 25 recessive, homozygous-viable alleles at the mouse *agouti* locus. Genetics 160: 659-674.

102. Miosge, L. A., J. Blasioli, M. Blery and C. C. Goodnow, 2002 Analysis of an ethylnitrosourea-generated mouse mutation defines a cell intrinsic role of nuclear factor κB2 in regulating circulating B cell numbers. J. Exp. Med. 196: 1113-1119.

103. Moran, J. L., A. D. Bolton, P. V. Tran, A. Brown, N. D. Dwyer *et al.*, 2006 Utilization of a whole genome SNP panel for efficient genetic mapping in the mouse. Genome Res. 16: 436-440.

104. Nam, Y. Y., J. K. Kim, D.-S. Cha, J.-W. Cho, K.-H. Cho *et al.*, 2006 A novel missense mutation in the mouse hairless gene causes irreversible hair loss: genetic and molecular analyses of $Hr^{m1ENU}$. Genomics 87: 520-526.

105. Paffenholz, R., R. A. Bergstrom, F. Pasutto, P. Wabnitz, R. J. Munroe *et al.*, 2004 Vestibular defects in head-tilt mice result from mutations in *Nox3*, encoding and NADPH oxidase. Genes Dev. 18: 486-491.

106. Papathanasiou, P., A. C. Perkins, B. S. Cobb, R. Ferrini, R. Sridharan *et al.*, 2003 Widespread failure of hematolymphoid differentiation caused by a recessive niche-filling allele of the Ikaros transcription factor. Immunity 19: 131-144.

107. Parkinson, N., R. E. Hardisty-Hughes, H. Tateossian, H.-T. Tsai, D. Brooker *et al.*, 2006 Mutation at the *Evi1* locus in *Junbo* mice causes susceptibility to otitis media. PLoS Genet. 2: e149.

108. Pask, A. J., H. Kanasaki, U. B. Kaiser, P. M. Conn, J. A. Janovick *et al.*, 2005 A novel mouse model of hypogonadotrophic hypogonadism: *N*-ethyl-*N*-nitrosourea-induced gonadotrophin releasing hormone receptor gene mutation. Mol. Endocrinol. 19: 972-981.

109. Pearce, S. R., J. Peters, S. Ball, M. J. Morgan, J. I. H. Walker *et al.*, 1995 Sequence characterization of ENU-induced mutants of glucose phosphate isomerase in mouse. Mamm. Genome 6: 858-861.

110. Peters, J., S. J. Andrews, J. F. Loutit and J. B. Clegg, 1985 A mouse β-globin mutant that is an exact model of hemoglobin Rainer in man. Genetics 110: 709-721.

111. Peters, T., C. Thaete, S. Wolf, A. Popp, R. Sedlmeier *et al.*, 2003 A mouse model for cystinuria type I. Hum. Mol. Genet. 12: 2109-2120.

112. Pinto, L. H., M. H. Vitaterna, K. Shimomura, S. M. Siepka, E. L. McDearmon *et al.*, 2005 Generation, characterization, and molecular cloning of the *Noerg-1* mutation of rhodopsin in the mouse. Vis. Neurosci. 22: 619-629.

113. Popp, R. A., E. G. Bailiff, L. C. Skow, F. M. Johnson and S. E. Lewis, 1983 Analysis of a mouse α-globin gene mutation induced by ethylnitrosourea. Genetics 105: 157-167.

114. Pretsch, W., B. Chatterjee, J. Favor, S. Merkle and R. Sandulache, 1998 Molecular, genetic and biochemical characterization of lactate dehydrogenase-A enzyme activity mutations in *Mus musculus*. Mamm. Genome 9: 144-149.

115. Rajaraman, S., W. S. Davis, A. Mahakali-Zama, H, K, Evans, L. B. Russell *et al.*, 2002 An allelic series of mutations in the *Kit ligand* gene of mice. I. Identification of point mutations in seven ethylnitrosourea-induced $Kitl^{Steel}$ alleles. Genetics 162: 331-340.

116. Rhodes, C. R., N. Parkinson, H. Tsai, D. Brooker, S. Mansell *et al.*, 2003 The homeobox gene *Emx2* underlies middle ear and inner ear defects in the deaf mouse mutant pardon. J. Neurocytol. 32: 1143-1154.

117. Rhodes, C. R., R. Hertzano, H. Fuchs, R. E. Bell, M. Hrabé de Angelis *et al.*, 2004 A *Myo7a* mutation cosegregates with stereocilia defects and low-frequency hearing impairment. Mamm. Genome 15: 686-697.

118. Ruan, H.-B., N. Zhang and X. Gao, 2005 Identification of a novel point mutation of mouse proto-oncogene *c-kit* through *N*-ethyl-*N*-nitrosourea mutagenesis. Genetics 169: 891-831.

119. Rudelius, M., A. Osanger, S. Kohlmann, M. Augustin, G. Piontek *et al.*, 2006 A missense mutation in the WD40 domain of murine Lyst is linked to severe progressive Purkinje cell degeneration. Acta Neuropathol. 112:267-276.

120. Runkel, F., A. Marquardt, C. Stoeger, E. Kochmann, D. Simon *et al.*, 2004 The dominant alopecia phenotypes Bareskin, Rex-denuded, and Reduced Coat 2 are caused by mutations in *gasdermin 3*. Genomics 84: 824-835.

121. Runkel, F., H. Büssow, K. L. Seburn, G. A. Cox, D. M. Ward *et al.*, 2006 *Grey*, a novel mutation in the murine *Lyst* gene, causes the *beige* phenotype by skipping of exon 25. Mamm. Genome 17: 203-210.

122. Rutschmann, S., K. Hoebe, J. Zalevsky, X. Du, N. Mann *et al.*, 2006 *PanR1*, a dominant negative missense allele of the gene encoding TNF-α (*Tnf*), does not impair lymphoid development. J. Immunol. 176: 7525-7532.

123. Sagai, T., H. Masuya, M. Tamura, K. Shimizu, Y. Yada *et al.*, 2004 Phylogenetic conservation of limb-specific, *cis*-acting regulator of Sonic hedgehog (*Shh*). Mamm. Genome 15: 23-34.

124. Sandberg, M. L., S. E. Sutton, M. T. Pletcher, T. Wiltshire, L. M. Tarantino *et al.*, 2005 c-Myb and p300 regulate hematopoietic stem cell proliferation and differentiation. Dev. Cell 8: 153-166.

125. Sanders, S., D. P. Smith, G. A. Thomas and E. D. Williams, 1997 A glucose-6-phosphate dehydrogenase (G6PD) splice site consensus sequence mutation associated with G6PD enzyme deficiency. Mutat. Res. 374: 79-87.

126. Sandulache, R., W. Pretsch, B. Chatterjee, W. Gimbel, J. Graw *et al.*, 1994 Molecular analysis of four lactate dehydrogenase-A mutants in the mouse. Mamm. Genome 5: 777-780.

127. Schneider, B., P. Hanke, W. Jagla, S. Wattler, M. Nehls *et al.*, 2004 Synergistic interaction of two independent genetic loci causes extreme elevation of serum IgA in mice. Genes Immun. 5: 375-380.

128. Schumacher, A., C. Faust and T. Magnuson, 1996 Positional cloning of a global regulator of anterior-posterior patterning in mice. Nature 383: 250-253.

129. Shima, N., S. A. Hartford, T. Duffy, L. A. Wilson, K. J. Schimenti *et al.*, 2003 Phenotype-driven identification of mouse chromosome instability mutants. Genetics 163: 1031-1040.

130. Sinha, D., M. K. Wyatt, R. Sarra, C. Jaworski, C. Slingsby *et al.*, 2001 A temperature-sensitive mutation of *Crygs* in the murine *Opj* cataract. J. Biol. Chem. 276: 9308-9315.

131. Skinner, J. A., B. M. Cattanach and J. Peters, 2002 The imprinted oedematous-small mutation on mouse chromosome 2 identifies new roles for Gnas and Gnasxl in development. Genomics 80: 373-375.

132. Smyth, I., X. Du, M. S. Taylor, M. J. Justice, B. Beutler *et al.*, 2004 The extracellular matrix gene *Frem1* is essential for the normal adhesion of the embryonic epidermis. Proc. Natl. Acad. Sci. USA 101: 13560-13565.

133. Steele, E. C., Jr., S. Kerscher, M. F. Lyon, P. H. Glenister, J. Favor *et al.*, 1997 Identification of a mutation in the MP19 gene, *Lim2*, in the cataractous mouse mutant *To3*. Mol. Vis. 3: 5.

134. Steele, E. C., Jr., M. F. Lyon, J. Favor, P. V. Guillot, Y. Boyd *et al.*, 1998 A mutation in the connexin 50 (Cx50) gene is a candidate for the No2 mouse cataract. Curr. Eye Res. 17: 883-889.

135. Steingrímsson, E., J. Favor, A. F. Ferré-D'Amaré, N. G. Copeland and N. A. Jenkins, 1998 $Mitf^{mi\text{-}enu122}$ is a missense mutation in the HLH dimerization domain. Mamm. Genome 9: 250-252.

136. Su, L.-K., K. W. Kinzler, B. Vogelstein, A. C. Preisinger, A. R. Moser *et al.*, 1992 Multiple intestinal neoplasia caused by a mutation in the murine homolog of the APC gene. Science 256: 668-670.

137. Tabeta, K., P. Georgel, E. Janssen, X. Du, K. Hoebe *et al.*, 2004 Toll-like receptors 9 and 3 as essential components of innate immune defense against mouse cytomegalovirus infection. Proc. Natl. Acad. Sci. USA 101: 3516-3521.

138. Tabeta, K., K. Hoebe, E. M. Janssen. X. Du, P. Georgel *et al.*, 2006 The *Unc93b1* mutation 3d disrupts exogenous antigen presentation and signaling via Toll-like receptors 3, 7 and 9. Nat. Immunol. 7: 156-164.

139. Thaung, C., K. West, B. J. Clark, L. McKie, J. E. Morgan *et al.*, 2002 Novel ENU-induced eye mutations in the mouse: models for human eye disease. Hum. Mol. Genet. 11: 755-767.

140. Timmer, J. R., T. W. Mark, K. Manova, K. V. Anderson and L. Niswander, 2005 Tissue morphogenesis and vascular stability require the Frem2 protein, product of the mouse myelencephalic blebs gene. Proc. Natl. Acad. Sci. USA 102: 11746-11750.

141. Toye, A. A., L. Moir, A. Hugill, L. Bentley, J. Quarterman *et al.*, 2004 A new mouse model of type 2 diabetes, produced by *N*-ethyl-nitrosourea mutagenesis, is the result of a missense mutation in the glucokinase gene. Diabetes 53: 1577-1583.

142. Traka, M., K. L. Seburn and B. Popko, 2006 *Nmf11* is a novel ENU-induced mutation in the mouse glycine receptor alpha 1 subunit. Mamm. Genome 17: 950-955.

143. Tsai, H., R. E. Hardisty, C. Rhodes, A. E. Kiernan, P. Roby *et al.*, 2001 The mouse *slalom* mutant demonstrates a role for Jagged1 in neuroepithelial patterning in the organ of Corti. Hum. Mol. Genet. 10: 507-512.

144. Tsai, T.-F., K.-S. Chen, J. S. Weber, M. J. Justice and A. L. Beaudet, 2002 Evidence for translational regulation of the imprinted *Snurf-Snrpn* locus in mice. Hum. Mol. Genet. 11: 1659-1668.

145. Van Agtmael, T., U. Schlötzer-Schrehardt, L. McKie, D. G. Brownstein, A. W. Lee *et al.*, 2005 Dominant mutations of Col4a1 result in basement membrane defects which lead to anterior segment dysgenesis and glomerulopathy. Hum. Mol. Genet. 14: 3161-3168.

146. Vinuesa, C. G., M. C. Cook, C. Angelucci, V. Athanasopoulos, L. Rui *et al.*, 2005 A RING-type ubiquitin ligase family member required to repress follicular helper T cells and autoimmunity. Nature 435: 452-459.

147. Vreugde, S., A. Erven, C. J. Kros, W. Marcotti, H. Fuchs *et al.*, 2002 Beethoven, a mouse model for dominant, progressive hearing loss DFNA36. Nat. Genet. 30: 257-258.

148. Vrijens, K., S. Thys, M. T. De Jeu, A. A. Postnov, M. Pfister *et al.*, 2006 *Ozzy*, a *Jag1* vestibular mouse mutant, displays characteristics of Alagille syndrome. Neurobiol. Dis. 24: 28-40.

149. Washington, J. L., III, D. Pitts, C. G. Wright, L. C. Erway, R. R. Davis *et al.*, 2005 Characterization of a new allele of Ames waltzer generated by ENU mutagenesis. Hear. Res. 202: 161-169.

150. Wen, B. G., M. T. Pletcher, M. Warashina, S. H. Choe, N. Ziaee *et al.*, 2004 Inositol (1,4,5) trisphosphate 3 kinase B controls positive selection of T cells and modulates Erk activity. Proc. Natl. Acad. Sci. USA 101: 5604-5609.

151. Wiltshire, T., M. T. Pletcher, S. Batalov, S. W. Barnes, L. M. Tarantino *et al.*, 2003 Genome-wide single-nucleotide polymorphism analysis defines haplotype patterns in mouse. Proc. Natl. Acad. Sci. USA 100: 3380-3385.

152. Wu, B., H. Mao, Y. Shao, Z. Xue and H. Li, 2003 Four kinds of ENU-induced white spot mice and chromosome locations of the mutant genes. Chin. Sci. Bull. 48: 2658-2664.

153. Wu, J.-Y., H.-J. Kao, S.-C. Li, R. Stevens, S. Hillman *et al.*, 2004 ENU mutagenesis identifies mice with mitochondrial branched-chain aminotransferase deficiency resembling human maple syrup urine disease. J. Clin. Invest. 113: 434-440.

154. Yu, P., R. Constien, N. Dear, M. Katan, P. Hanke *et al.*, 2005 Autoimmunity and inflammation due to a gain-of-function mutation in phospholipase C$\gamma$2 that specifically increases external $Ca^{2+}$ entry. Immunity 22: 451-465.

155. Yu, Q., Y. Shen, B. Chatterjee, B. H. Siegfried, L. Leatherbury *et al.*, 2004 ENU induced mutations causing congenital cardiovascular anomalies. Development 131: 6211-6223.

156. Zarbalis, K., S. R. May, Y. Shen, M. Ekker, J. L. R. Rubenstein *et al.*, 2004 A focused and efficient genetic screening strategy in the mouse: identification of mutations that disrupt cortical development. PLoS Biol. 2: e219.

157. Zdarsky, E., J. Favor and I. J. Jackson, 1990 The molecular basis of *brown*, an old mouse mutation, and of an induced revertant to wild type. Genetics 126: 443-449.

158. Zingg, B. C., W. Pretsch and H. W. Mohrenweiser, 1995 Molecular analysis of four ENU-induced triosephosphate isomerase null mutants in *Mus musculus*. Mutat. Res. 328: 163-173.

159. Zohn, I. E., Y. Li, E. Y. Skolnik, K. V. Anderson, J. Han *et al.*, 2006 p38 and a p38-interacting protein are critical for downregulation of E-cadherin during mouse gastrulation. Cell 125: 957-969.

160. Zoltewicz, J. S., N. J. Stewart, R. Leung and A. S. Peterson, 2004 Atrophin 2 recruits histone deacetylase and is required for the function of multiple signaling centers during mouse embryogenesis. Development 131: 3-14.

161. Zwingman, T. A., P. E. Neumann, J. L. Noebels and K. Herrup, 2001 Rocker is a new variant of the voltage-dependent calcium channel gene *Cacna1a*. J. Neurosci. 21: 1169-1178.
